# Pseudogenes in Cardiovascular Disease

**DOI:** 10.3389/fmolb.2020.622540

**Published:** 2021-02-10

**Authors:** Yanyan Qi, Xi Wang, Wenbo Li, Dongchang Chen, Hua Meng, Songtao An

**Affiliations:** ^1^Department of Cardiology, Anesthesiology and Emergency Medicine, Henan Province People's Hospital and People's Hospital of Zhengzhou University, Zhengzhou, China; ^2^Department of Cardiology, People's Hospital of Zhengzhou University, Zhengzhou, China; ^3^Department of Cardiology, Henan Province People's Hospital and People's Hospital of Zhengzhou University, Zhengzhou, China

**Keywords:** cardiovascular disease, pseudogenes, non-coding gene, mutation, protein-coding, gene regulation

## Abstract

Cardiovascular disease is the main disease that affects human life span. In recent years, the disease has been increasingly addressed at the molecular levels, for example, pseudogenes are now known to be involved in the pathogenesis and development of cardiovascular diseases. Pseudogenes are non-coding homologs of protein-coding genes and were once called “junk gene.” Since they are highly homologous to their functional parental genes, it is somewhat difficult to distinguish them. With the development of sequencing technology and bioinformatics, pseudogenes have become readily identifiable. Recent studies indicate that pseudogenes are closely related to cardiovascular diseases. This review provides an overview of pseudogenes and their roles in the pathogenesis of cardiovascular diseases. This new knowledge adds to our understanding of cardiovascular disease at the molecular level and will help develop new biomarkers and therapeutic approaches designed to prevent and treat the disease.

## Introduction

Cardiovascular diseases (CVDs) are the leading cause of death, taking an about 18 million lives globally every year (Goradel et al., 2018). CVDs are composed of coronary heart disease, cerebrovascular disease, rheumatic heart disease as well as other related conditions. A better understanding of CVDs at the molecular level is particularly important to develop preventive and therapeutic strategies. In this regard, tremendous effects have been made toward the elucidation of molecular mechanisms underlying various CVDs (Uchida and Dimmeler, 2015; Li S. et al., 2018). For example, expression of long non-coding RNAs (lncRNAs) has been detected under normal physiological conditions and in disease states, some lncRNAs are found to regulate acute myocardial infarction (such as Novlnc6) (Uchida and Dimmeler, 2015) and acute heart failure [such as Mhrt (Zhang et al., 2019), H19 (Greco et al., 2016), and growth arrest specific 5 (Wang G. et al., 2019)], and gene transcripts such as MALAT1 and Tie-1-AS are found to control the growth and functions of blood vessels (Uchida and Dimmeler, 2015). Recently, Spinraza (a novel antisense oligonucleotide therapy) is approved by FDA to treat spinal muscular atrophy resulted from pseudogene SMN2, in which the antisense oligonucleotide is used to increase SMN2 exon 7 inclusion, thus increasing levels of survival motor neuron (SMN) protein (Chiriboga, 2017; Paton, 2017; Berciano et al., 2020). This is an excellent example of how our understanding of molecular mechanism of pseudogene has been translated to important therapeutic solution.

Pseudogene was first identified in 1977 (Jacq et al., 1977), and generally refers to non-functional DNA sequences derived from non-sense or frameshift mutations on protein coding regions of

ancient functional genes. Therefore, it has high sequence homolog with the parental gene but does not encode any specific protein or peptide. Most pseudogenes in the human genome have not been characterized for biological functions. For a long time, they have been considered as “junk gene” as a result of ongoing evolution. However, data obtained during the past decade have indicated that this interpretation of the usefulness of pseudogenes is not entirely correct, and many pseudogenes have important biological and genetic functions (Jacq et al., 1977; Jingsi et al., 2015; Xie et al., 2019; Cheetham et al., 2020). With the advances in sequencing technology, more and more pseudogenes were identified (Dong et al., 2016). In the review, we address the classification, identification and role of pseudogenes in the pathogenesis of CVDs with emphasis on the mechanisms underlying gene expression regulation by pseudogenes.

## Classification of Pseudogenes

**Figure 1:**
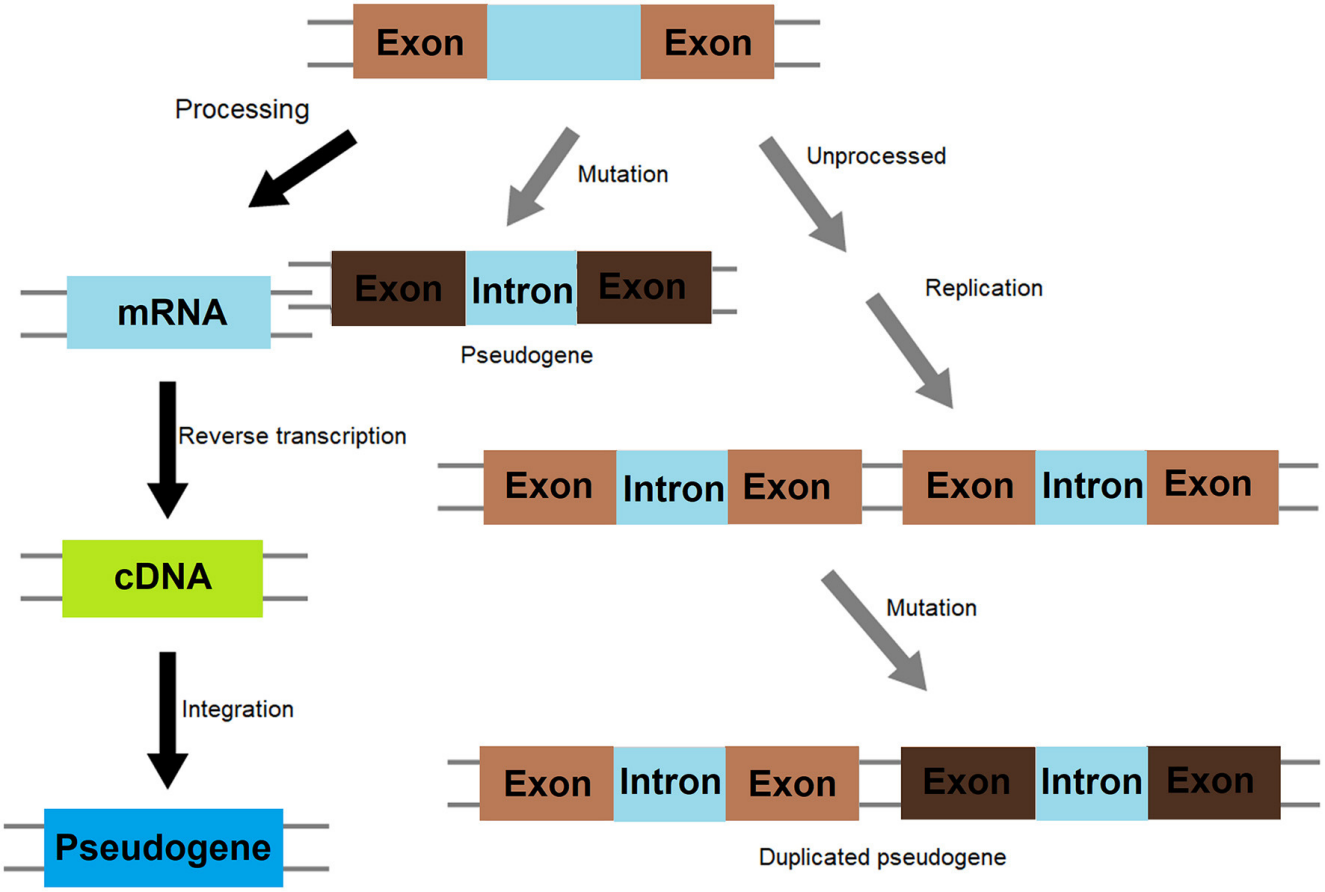
Molecular mechanism of pseudogene formation.

Pseudogenes were originally defined as non-functional genomic DNA sequences that are initially derived from genes. Therefore, it is assumed that various kinds of gene mutations occurred to generate pseudogenes are selectively neutral and the mutated genes are able to transmit to the next generations (Balakirev and Ayala, 2003). Generally speaking, pseudogenes are highly homologous with DNA sequences of ancestral functional genes but cannot be translated into protein due to the lack of critical regulatory elements such as promoters or the presence of premature stop codons resulted from frameshift mutations. They can be divided into non-processed or processed pseudogenes (Figure 1) (Milligan et al., 2016; Maranda et al., 2019). Non-processed pseudogenes are mainly derived from functional genes by processes such as unequal crossing over events during cell division. In these events, the homologous sequences are not paired precisely, a sequence is deleted in one strand and replaced with a duplication from its sister chromatid in mitosis or from its homologous chromosome during meiosis. They generally contain a promoter and other regulatory sequence elements such as enhancers and an intact or partial exon-intron structure. Unitary pseudogenes are a class of unprocessed pseudogenes that do not have functional counterparts in the genome (Zhang et al., 2010) and can be identified by analyzing the global inventory of orthologs between the human genome and its mammalian relatives (Zhang et al., 2010). On the other hand, processed pseudogenes result from the insertion of reversely-transcribed and mature RNA molecule (intron-less cDNA) into the genome, and the abundance of processed pseudogenes are therefore positively related to the expression level of genes from which the RNA molecules are derived (McDonell and Drouin, 2012). They usually do not have introns but a poly-A tail as cDNA sequences (Weiner et al., 1986) and are preferentially located in regions of low recombination rates in the human genome (Liu et al., 2010). They may be formed as a result of somatic reactivation of retrotransposons occurring during cancer development (Cooke et al., 2014). Different from processed pseudogenes, the presence of genetic structures in non-processed pseudogenes, such as exon-intron structures, may provide clues to trace the origin of these genes through bioinformatics analysis. Evolutionally, processed pseudogenes are derived from functional homologs and have distinct genetic features stemming from point mutations, deletions, and insertions. Once formed, pseudogenes can be inherited and transmitted to offspring and generate secondary pseudogenes through evolution.

## Identification of Pseudogene

With the development of computer technology, bioinformatics, and especially sequencing technology, the whole genome sequences of many species have been sequenced and published. Pseudogenes are being identified through sequence alignment and genome-wide transcriptomic and proteomic studies (Xu and Zhang, 2016). Using RNA sequencing (RNA-seq), new transcriptomes are becoming available quickly. The transcriptomes can be assessed to discover pseudogenes using bioinformatics tools to search, compare, and screen relevant genomic databases and proteomic databases, and estimate the types and abundance of pseudogenes. Several tools are currently available for the identification of pseudogenes, such as PseudoPipe (Zhang et al., 2006) (http://www.pseudogene.org/pseudopipe/), Pseudofinder (https://github.com/filip-husnik/pseudo-finder), Retrofinder, and REtrotransposed Gene EXPlorer (Regexp) (Molineris et al., 2010). PseudoPipe is a computational pipeline based on homology to screen and search a mammalian genome. It can identify both non-processed and processed pseudogene sequences. In this program, the sequences of candidate pseudogene sequences are compared with protein sequences to remove annotated gene sequences and repetitive redundant sequences, generating clusters of genes from a unique parent. Pseudogenes are also grouped based on their homology, intron-exon structures, and presence of stop codons and frameshifts in the sequences (Zhang et al., 2006). Different from PseudoPipe, Pseudofinder is designed to detect pseudogene candidates from bacterial and archaeal genomes.

Despite purposes and procedures, all these methods depend on genomic, transcriptomic and proteomic data, and are very efficient for species with expressed sequence tags (EST) or proteomic data. However, they are not effective for species whose information is not available or organisms lacking large EST collections. REGEXP was developed to systematically identify retrotransposition events. This method, unlike other existing approaches, does not depend on a prior knowledge of mRNA sequences. Using this pipeline, 2,288 processed pseudogenes have been identified in the human genome (Molineris et al., 2010). Recently, several new approaches have been developed to identify pseudogenes from various sources. For examples, competing endogenous RNAs (ceRNAs) networks are explored to link pseudogenes and circular RNAs (circRNAs) to identify pseudogenes, which also provide significant insights into gene regulatory network with implication of human diseases (Li et al., 2018a). In addition, a computational pipeline (CIRCpseudo) has been developed to identify potential pseudogenes derived from circRNAs based on the feature that a circRNA-derived pseudogene would have an exon-exon junction in a reversed orientation (Dong et al., 2016). After searching the mouse and human reference genomes, 42 pseudogenes originated from circRFWD2 in mouse and 24 circRNA-derived pseudogenes in human were identified (Dong et al., 2016). Several databases have been developed to provide information about the transcriptional regulation, expression, functions, and mechanisms of pseudogenes as well as their roles in biological processes and diseases (Khelifi et al., 2005; Zheng et al., 2018). In addition, circRNAs have also been implicated in many diseases, including tumor and heart disease (Kalyana-Sundaram et al., 2012; Wang et al., 2016; Li et al., 2018b).

## Function of Pseudogene

Pseudogenes cannot be translated into proteins, so they were once considered to be “dead genes” or “junk genes.” Although intensive efforts and studies have been made to elucidate and understand the function of pseudogenes, their biological and genetic roles still remain largely unknown. However, during the past decades, functional studies of pseudogenes have evolved from the initial discovery of single pseudogene to the modes and mechanisms of parental gene regulation by pseudogenes. It is found that pseudogenes regulate parental genes mainly via their transcripts. Expressed pseudogenes may be transcribed to form antisense RNAs, which may complement with the transcripts of parental genes to generate double strands or complemented stands, leading to change in expression of parent genes or resulting in destabilization of mRNA from the parental genes. For example, in an insertion-generated mouse mutant having polycystic kidneys and exhibiting bone deformity, the introduced transgene was found to have inserted in the vicinity of an expressed pseudogene Makorin1, termed Makorin1-p1. This transgenic insertion decreased the transcription level of Makorin1-p1, leading to destabilization of cellular Makorin1 mRNA (Hirotsune et al., 2003). Pseudogenes may form endogenous small interfering (SI) RNAs, when the double strand RNAs between pseudogene transcripts and their parent transcripts or repetitive sequences of pseudogene are sliced by endonuclease. These siRNAs may control and regulate the expression of their parental genes through mechanisms such as RNA interference (Abedini et al., 2018). For instance, it was found that many endogenous siRNAs (endo-siRNAs) are usually produced and formed from RNAs such as double-stranded RNAs generated by hybridization of transcripts spliced from genes that can code protein to antisense transcripts from homologous pseudogenes. Endo-siRNAs can interact with Piwi (P-element-induced wimpy testis)-interacting RNAs to repress the movement of transposable genetic elements (Tam et al., 2008). In addition, the competitive endogenous RNA (ceRNA) may compete with miRNA generated by pseudogenes for target genes to regulate the expression of target genes (Thomson and Dinger, 2016). For example, pseudogene RNAs can function as “sponge” to competitively bind miRNAs, leading to release or attenuation of repression through separating targeted miRNAs away from parental mRNAs, since binding of miRNA to the target RNA is not 100% complementary, it is likely that one miRNA can bind to multiple target RNAs. Such interactions and binding generate a complex gene regulatory network that may affect the expression of functional genes. An increase in abundance of pseudogene RNA can strengthen the blocking of miRNA and eliminate suppression on the expression of parental genes, leading to an up-regulation of parental gene expression. Studies have shown that the regulation of gene expression by pseudogene in this manner needs the presence of RNAs that bear common and functional metal regulatory elements (MREs) (Poliseno et al., 2010). Furthermore, some pseudogenes can also encode short peptides and proteins, although this is contradictive to original definition of pseudogenes. Some pseudogenes may gain or retain function during evolution. For example, the processed pseudogene NLRP2P is related specifically to higher primate. It is found to be closely associated with the Pyrin-only protein 2 (POP2/PYDC2). The open-reading frame of the NLRP2P gene located on chromosome X has characteristics that are consistent with a processed pseudogene (retrotransposon). However, it encodes Pyrin-domain-related protein with 45-amino acids (Porter et al., 2014). Using a new bioinformatics method, it was found that 40% lncRNAs and pseudogene RNAs expressed in human cells are translated into peptides and 74% of pseudogene peptides have conserved ORFs in mouse transcripts, implying that these peptides are potentially functional (Ji et al., 2015). As a consequence of biomedicine research, large number of pseudogenes have been implicated in many human diseases (Pink et al., 2011; Grander and Johnsson, 2016), including cancer (Emadi-Baygi et al., 2017; Tutar et al., 2018) and CVDs (Table 1).

**Table 1:** Pseudogenes and circRNAs identified in cardiovascular disease.

**Disease**	**Pseudogene/circRNA**	**Function**	**Mechanism**	**References**
Atherosclerosis	APOOP1	Increasing low-density lipoprotein cholesterol		Montasser et al., 2018
	CLRX.1/NOD24 (NLRP2P)	Impairing NF-κB p65 transactivation		Porter et al., 2014
	Lp(a)-like 2	Elevating serum lipid		Wu et al., 2019; Chen et al., 2020
	RPL38 processed pseudogene			Dang et al., 2017; Tan et al., 2017
	SAA3	Stimulates vascular proteoglycan synthesis		Wilson et al., 2008; Thompson et al., 2018
	ANRIL	Reducing vascular EC apoptosis	Protein binding	Song et al., 2017
	Circ_0124644	Inducing vascular endothelium injury	Unknown	Zhao et al., 2017; Zhang et al., 2020
	Circ-ANRIL		Protein binding	Chen et al., 2020
	Hsa_circ_0010729	Reducing apoptosis	mRNA sponge	Dang et al., 2017
	circHIPK3	Improving cell viability	mRNA sponge	Shan et al., 2017
Myocardial infarction	Carboxylesterase 1 pseudogene 1			Zhang et al., 2016; Koseler et al., 2020
	CDR1AS		mRNA sponge	Zhang et al., 2016
	ZFAS1		mRNA sponge	Zhang et al., 2016
	MICRA		mRNA sponge	Salgado-Somoza et al., 2017
	HRCR	Inhibiting cardiac hypertrophy	mRNA sponge	Wang et al., 2016
	MFACR	Reducing cardiomyocyte cell apoptosis	mRNA sponge	Wang et al., 2017
	Circ-Amotl1		Protein binding	Zeng et al., 2017
Myocardial fibrosis	Circ_010567	Targeting TGF-beta1	mRNA sponge	Zhou and Yu, 2017
Cardiomyopathy	Foxo3	Interacting with anti-senescent proteins	Protein binding	Du et al., 2017
	CircTtn	Activating the *IGF2*/phosphatidylinositol 3-kinase *(PI3K)/AKT* signaling pathway.	mRNA sponge	van Heesch et al., 2019; Wang X. et al., 2019
Pulmonary arterial hypertension	hsa_circ_0016070	Vascular remodeling	mRNA sponge	Zhou et al., 2019
	hsa_circ_0022342 hsa_circ_0002062	Inducing apoptosis in aortic smooth muscle cells	Regulating gene expression biomarkers	Miao et al., 2017
Aortic aneurysm	Circ-000595		mRNA sponge	Zheng et al., 2015
Aortic dissection	Circ-101238		mRNA sponge	Duggirala et al., 2015; Cheng et al., 2020
Cardiac hypertrophy	Circ-000203	Pro-hypertrophic effect	Suppressing gene expression	Li et al., 2020

## Pseudogenes and CVDs

### Atherosclerosis

Statins are the most commonly prescribed CVD drugs. To identify biomarkers and determinants that are responsible for statin response, Kim et al. compared whole transcriptome sequence data of patients collected from simvastatin and control after simvastatin treatment. They found that one of the most differentially expressed genes is zinc finger protein 542 pseudogene (ZNF542P). It is considered as the signature gene because the changes of its expression are most correlated with statin-induced changes in the cellular level of cholesterol ester. They further showed that knock-down of the ZNF542P gene in a human hepatoma cell line increases the intracellular levels of cholesterol ester after the cells were exposed to simvastatin. These findings indicate that ZNF542P may have a role in low-density lipoprotein cholesterol (LDL-C) response to simvastatin (Kim et al., 2018). However, since these genes were identified from cell lines, not directly from tissues of patients treated with simvastatin, it is likely that they might not be the most responsive genes *in vivo*.

Elevated LDL-C level is a main risk factor for CVDs because it contributes to, and enhances the development and progression of atherosclerotic lesions. Currently, only 20% of the variation in LDL-C levels can be attributed to genetic variants. Through an array-based association analysis, it was found that a variant is strongly associated with LDL-C levels and the expression of a transcribed pseudogene, APOOP1 located on chromosome 5, increased LDL-C level and vascular plaque formation (Montasser et al., 2018), suggesting that this may be a novel mechanism of lipid homeostasis.

Serum amyloid-alpha (SAA) and high-sensitivity C-reactive protein (hs-CRP) are sensitive biomarkers of acute inflammation and are related to atherosclerosis. Recent studies show that there is a significant and independent relationship between SAA and development of potential cardiovascular events, suggesting that systemic inflammations, manifested as high SAA or hs-CRP levels, may accelerate the destabilization of existing atherosclerotic plaques (Johnson et al., 2004). SAA has three transcripts, SAA1/SAA2/SAA3. Among them, SAA3 is a human pseudogene. In the animal model, it is an expressed acute phase isoform in mice when acute inflammatory reaction occurs. Using ApoE(–/–) mouse model, it was shown that over-expression of SAA3 results in a 4-fold increase in atherosclerosis lesion size over control while knockdown of SAA3 decreases atherosclerosis (Johnson et al., 2004), implying that this gene may be a new therapeutic target for atherosclerosis.

Non-coding locus INK4 on chromosome 9p21.3 is closely related to atherosclerosis. The antisense non-coding RNA in INK4 locus *ANRIL* was confirmed to be involved in the pathological process of atherosclerosis, leading to carotid plaque, stroke, aneurysms, peripheral arterial disease, heart failure, and cardiovascular death events (Holdt and Teupser, 2012). However, *ANRIL* is not directly related to high risk factors such as hypertension and hyperlipidemia. Mechanistically, it forms cicr*ANRIL*, which binds to a number of proteins such as the nucleolar protein pescadillo homolog 1 (PES1), leading to impairment of exonuclease-mediated pre-rRNA processing and ribosome biogenesis in vascular smooth muscle cells and macrophages, resulting in apoptosis and inhibition of proliferation (Holdt et al., 2016).

### Idiopathic Pulmonary Arterial Hypertension

**Figure 2:**
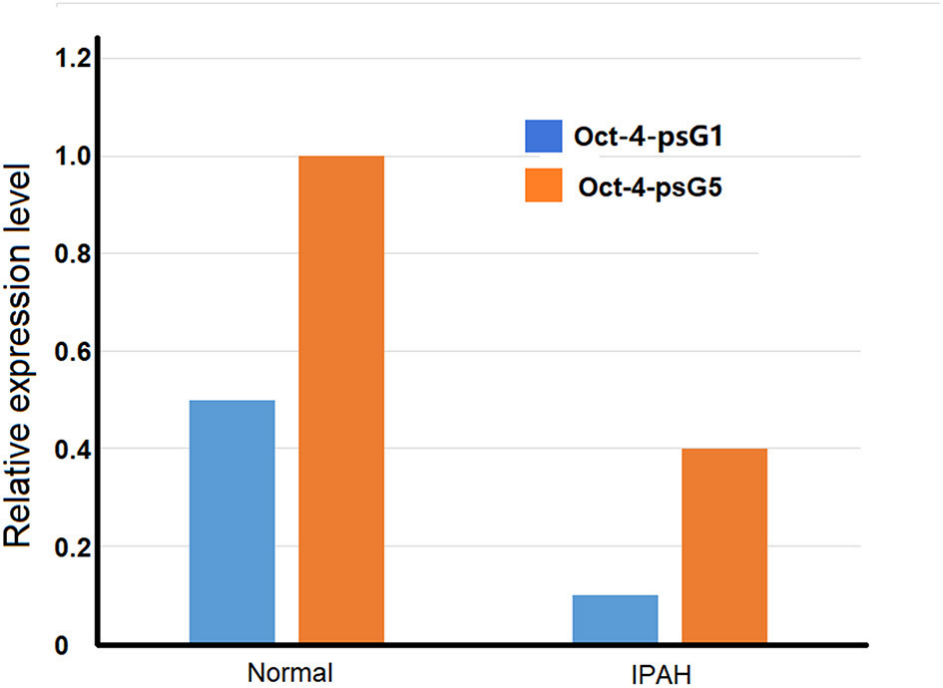
Comparison of expression of Oct-4 pseudogenes PSG1 and PSG5 in pulmonary arterial smooth muscle cells (PASMC) from idiopathic pulmonary arterial hypertension (IPAH) patients and normal individuals.

Idiopathic pulmonary arterial hypertension (IPAH) is a vasculopathy. It is characterized by increased pulmonary vascular resistance due to vasoconstriction and/or lung remodeling defects such as plexiform lesions, which are the hallmark of the PAH, as well as massive cell proliferation, remarkable, and irreversible vascular and angiogenic dysfunction. Octamer-binding transcription factor 4 (Oct4) is a transcription factor and is used as pluripotency markers in embryonic stem cell research. Six pseudogenes of the Oct4 family have been identified by using bioinformatics approach to analyze the genomic nucleotide sequences. Some of them are found involved in the excessive proliferation of pulmonary arterial smooth muscle cells (PASMC) in patients with IPAH. Therefore, they may have a role in the pathogenesis and development of IPAH. The expressions of Oct-4 isoforms are upregulated in IPAH-PASMC conditions. However, the mRNA levels of Oct-4 pseudogene Oct-4-psG1 and Oct-4-psG5 are significantly down-regulated in IPAH-PASMC (Figure 2) (Firth et al., 2010).

### Heart Failure

Heart failure (HF) is a heterogeneous clinical syndrome stemming from cardiac overload and injury. The disease is expected to increase steadily in the upcoming years due to demographic changes such as aging. Therefore, a better understanding of molecular mechanisms underlying the disease is crucial for prevention and treatment of the disease. Using RNA-Seq, the expression of lncRNAs in 22 transplanted human models of HF hearts was studies. In HF heart models, a total of 84,793 mRNA coding and non-coding transcripts were identified using RNA-Seq, including 13,019 protein-coding genes, 2,085 lncRNA genes, and 1,064 pseudogenes. Based on the Ensembl release 73 (Genome Reference Consortium human genome build 37), 48 lncRNAs, 27 pseudogenes, and 30 antisense RNAs were identified for a total of 105 differentially expressed lncRNAs in the HF hearts. Compared with the donor hearts, HF hearts showed that 7.7% of protein-coding genes, 3.7% of lncRNAs, and 2.5% of pseudogenes are differentially expressed. *In silico* functional analyses suggested that many of these pseudogenes have possible regulatory roles and are of important for future mechanistic study (Di Salvo et al., 2015a). Based on RNA sequencing, 800–1,000 differentially expressed genes (DEGs) were identified between HF right ventricular (RV) and unused donor human heart RVs (DON RV), and many of them are likely pseudogenes. Since these DEGs had high sensitivities, specificities, predictive values and areas under the receiver operating characteristic curves, they might be novel HF myocardial biomarkers (Di Salvo et al., 2015b). In these studies, the sequenced transcripts were categorized based on the Ensembl release 73, which was released in 2013. More deferentially expressed transcripts could be identified with newer versions of the Ensembl release. Furthermore, annotation of these transcripts with other databases, such as GRCh38.p13 (Genome Reference Consortium Human Build 38 patch release 13) may offer more functional and classification information. When RNA-seq is used to profile the transcripts, the RNA sources have great impact on the transcriptome. In their work, end-stage human myocardium was used for RNA-seq, which may generate sequence bias due to medications and therapies (Di Salvo et al., 2015b), resulting in biomarkers less suitable for non-end stage patients. In addition, *in vivo* and *in vitro* experiments are needed to defined and validate the biological functions of the genes identified in silica. In general, parental and coding genes have high expression levels than corresponding pseudogenes. However, some pseudogenes have been found highly expressed. For example, the expression level of pseudogene FBXO43 is far more abundant than the corresponding parental gene (Shoji et al., 2014). Endogenous siRNA produced from pseudogene FBXO43 does not target the transcript of FBXO43, but the transcripts related to FBXO43 such as the transcripts of MAPK1, MDM2, OSMR, and IRAK3 and NMRA-like protein NMRAL1, which is a redox-sensitive transcriptional regulator. Reduced expression of NMRAL1 increases nitric oxide (NO) production and reduces cell viability. Compared with unused donor hearts, the expression level of NMRAL1 pseudogenes, but not NMRAL1 in right ventricle of HF was significantly reduced, indicating that NMRAL1 pseudogenes are involved in HF in right ventricle (Garciandia and Suarez, 2013). Identifications of genes including pseudogenes would provide targets for future molecular analysis on HF.

### Other CVDs

The occurrence and prevalence of coronary artery disease are gender-depended. Men are more likely to be affected than women. Therefore, it is possible that the Y chromosome may contribute to this sexual difference. An analysis of 11 genetic markers positioned on the male-specific region of the Y chromosome in 3233 British men revealed nine haplogroups. The carriers of haplogroup I had a risk of coronary artery disease in about 50% higher likelihood than men with other Y chromosome lineages. The high risk possibly results from the interactions of immunity and inflammation in cardiac systems and associated with high blood pressure and myocardial infarction, but not with BMI, blood lipids, blood pressure, and C-reactive protein (Charchar et al., 2012). For men in the haplogroup I population, the elevated risk of coronary artery disease appears to be related to the down-regulations of several genes such as ubiquitously transcribed-tetratricopeptide repeat, the Y-linked gene (*UTY*) and protein kinases, and Y-linked, pseudogene (*PRKY*) in macrophages (Bloomer et al., 2013). *PRKY* is a transcribed pseudogene without exon 6 and a part of exon 5 (that encodes the functional domains of the kinase). However, the biological function of PRKY is largely unknown. Its functional homolog is located on the X chromosome and is involved in the maturation of macrophage and development of kidney (Li et al., 2002; Glesne and Huberman, 2006).

Dilated cardiomyopathy (DCM) and hypertrophic cardiomyopathy (HCM) are two major cardiomyopathies. Khan et al. compared circRNA expression profiles in DCM, HCM, and normal cardiac tissues and found that RNA-binding motif protein 20 (RBM20), an essential protein for normal splicing of many cardiac genes, is also crucial to generate of a subset of circRNAs from the I-band of the titin gene. In RBM20-null mice, these titin circRNAs are down-regulated in DCM but not in HCM (Khan et al., 2016). These cardiac circRNAs are mostly (~90%) produced from constitutive exons and less (~10%) from alternatively spliced exons and are generated at the expense of their linear counterpart (Aufiero et al., 2018).

Arrhythmogenic right ventricular cardiomyopathy (ARVC) is a family-heritable disorder. It is characterized by progressive degeneration and destabilization of right ventricular myocardium, increased incidence of arrhythmias and an increased risk of sudden death at a young age. To identified gene associated with the disease, a number of genes located in the critical region of the microsatellite markers were screened, including laminin receptor 1 pseudogene 6 *LAMR1P6*, which is a processed transposable element within the ARVC6 critical interval (Asano et al., 2004) and a functional *lamr1* retroposon gene is a cause of ARVC observed in the mouse via regulating mRNA stability of a homologous gene (Yonemura et al., 1998).

Marfan syndrome (MFS) is an autosomal dominant connective tissue disorder with an estimated incidence of 1/5,000 peoples. It affects the cardiovascular system as well as lung, skin, and dura. Since fibrillin-1 (FBN1) mRNA is indicator for MFS severity, identification of trans-acting regulators that controls FBN1 expression is important to elucidate the mechanism. Pseudogene, SNX7-ps1, is found to be associated with expression of a neighboring gene SLN (encoding sarcolipin) that plays role in skin fibroblasts. Since SNX7-ps1 expression is positively correlated with the SLN gene, it is likely that the two genes are controlled by regulatory elements for their expression (Benarroch et al., 2018).

**Figure 3:**
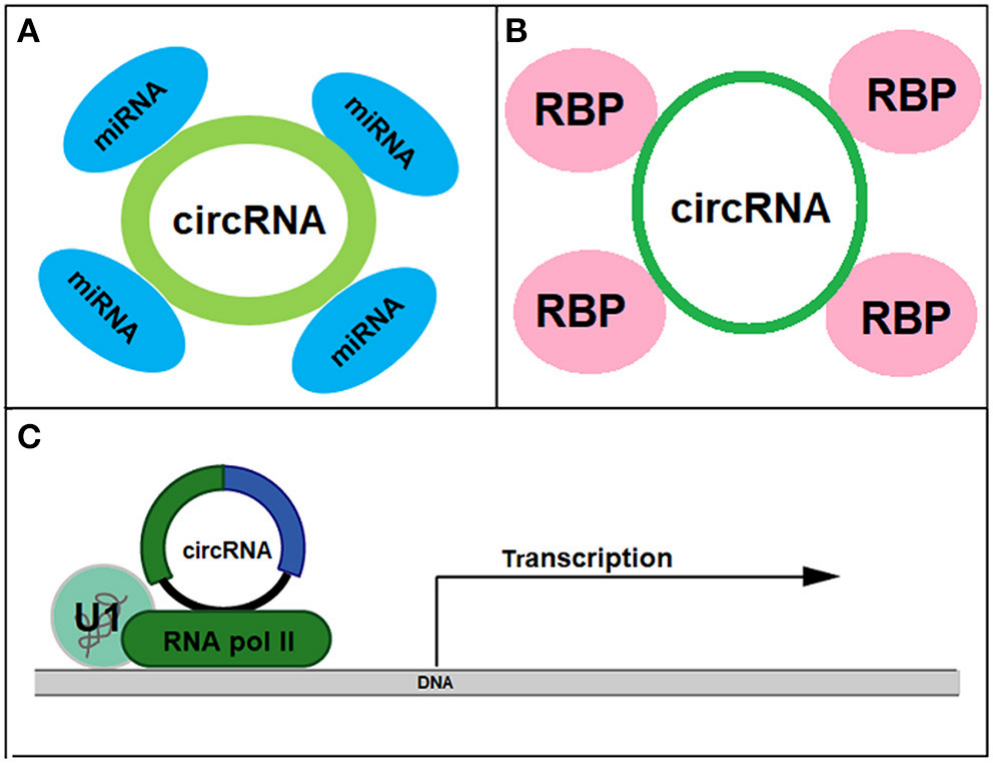
Biological functions of circRNAs as miRNA sponge **(A)**, protein sponge **(B)**, and transcriptional regulator **(C)**.

CircRNAs have been shown to involve in a number of CVD conditions ranging from atherosclerosis to cardiac hypertrophy (Table 1) and they function as miRNA and protein sponges or transcriptional regulators to exert biological functions (Figure 3). For instance, cicrFoxO3 derived from the *FoxO3* gene is highly expressed in the elderly and is positively corelated to senescence-associated β-galactosidase, SA-β-gal. It is proposed that cicrFoxO3 may be a protein sponge of inhibitor of differentiation-1, focal adhesion kinase, E2F transcription factor I, and hypoxia inducible factor 1, resulting in reduced availability of these proteins, thus reduced translocation of anti- senescence proteins into the nuclei and cellular senescence (Du et al., 2017). RBPs (RNA-binding proteins) have been found to be expressed and regulated in the heart. After treatment with doxorubicin, expression of circRNAs from *Ttn* (Titin), *Fhod3* (Formin homology 2 domain containing 3), and *Strn3* (Striatin, calmodulin-binding protein 3) and *Qki5* was down-regulated, leading to reduced cell viability and cardiac senescence (Gupta et al., 2018).

## Conclusion

With the continuous development of sequencing technology and bioinformatics, more and more pseudogenes are going to be discovered and studied. Current investigations have shown that pseudogenes can regulate gene expression and play important roles in the occurrence and development of CVDs. They may be used as new diagnostic markers or therapeutic targets to enhance our efforts to combat the diseases. However, compared to other molecular studies in human diseases, research on pseudogenes in CVDs is still in its infancy. More studies are needed to further our understanding of molecular mechanisms related to the role of pseudogenes in cardiovascular and other diseases.

## Author Contributions

YQ, XW, and SA designed the study. YQ, XW, WL, DC, and HM collected the data and performed analysis. WL, DC, HM, and SA drafted the manuscript. All authors read and approved the final manuscript.

## Conflict of Interest

The authors declare that the research was conducted in the absence of any commercial or financial relationships that could be construed as a potential conflict of interest.
